# In Situ N, O-Dually Doped Nanoporous Biochar Derived from Waste Eutrophic *Spirulina* for High-Performance Supercapacitors

**DOI:** 10.3390/nano13172431

**Published:** 2023-08-27

**Authors:** Yihao Geng, Jieni Wang, Xuanyu Chen, Qizhao Wang, Shuqin Zhang, Yijun Tian, Chenxiao Liu, Lin Wang, Zhangdong Wei, Leichang Cao, Jinglai Zhang, Shicheng Zhang

**Affiliations:** 1 Miami College, Henan University, Kaifeng 475004, China; gyh618@henu.edu.cn (Y.G.); jieniwang@126.com (J.W.); xuanyuchen@henu.edu.cn (X.C.); 17367066458@163.com (Q.W.); zhangshuqin@henu.edu.cn (S.Z.); 104753200976@henu.deu.cn (Y.T.); lcxdxyyx@henu.edu.cn (C.L.); wanglin@henu.edu.cn (L.W.); 15565139820@163.com (Z.W.); 2 College of Chemistry and Molecular Sciences, Henan University, Kaifeng 475004, China; zhangjinglai@henu.edu.cn; 3 Shanghai Key Laboratory of Atmospheric Particle Pollution and Prevention (LAP3), Department of Environmental Science and Engineering, Fudan University, Shanghai 200433, China; zhangsc@fudan.edu.cn

**Keywords:** algae biomass, nanoporous biochar, supercapacitor, energy storage

## Abstract

Sustainable and high-performance energy storage materials are crucial to address global energy and environmental challenges. In this study, *Spirulina platensis* was used as the carbon and nitrogen source, and *Spirulina*-based nanoporous biochar (SNPB) was synthesized through chemical activation using KOH as the activating agent in N_2_ atmosphere. SNPB-800-4 was characterized by N_2_ adsorption–desorption and XPS, showing a high specific surface area (2923.7 m^2^ g^−1^) and abundant heteroatomic oxygen (13.78%) and nitrogen (2.55%). SNPB-800-4 demonstrated an exceptional capacitance of 348 F g^−1^ at a current density of 1 A g^−1^ and a remarkable capacitance retention of 94.14% after 10,000 cycles at a current density of 10 A g^−1^ in 6 M KOH. Notably, symmetric supercapacitors SNPB-800-4//SNPB-800-4 achieved the maximum energy and power densities of 17.99 Wh kg^−1^ and 162.48 W kg^−1^, respectively, at a current density of 0.5 A g^−1^, and still maintained 2.66 Wh kg^−1^ when the power density was increased to 9685.08 W kg^−1^ at a current density of 30 A g^−1^. This work provides an easily scalable and straightforward way to convert waste algae biomass into in situ N, O-dually doped biochar for ultra-high-power supercapacitors.

## 1. Introduction

Persistent fossil fuel depletion and environmental pollution have necessitated the development of high-performance energy storage systems using sustainable feedstocks [1,2]. Supercapacitors, also known as ultracapacitors or electrochemical capacitors, have emerged as a promising alternative to traditional batteries because of their numerous advantages, including high energy density, high power density, fast charging and discharging rates, extended cycle life, excellent cycle stability, and reduced environmental impact [3,4,5]. According to their energy mechanisms, supercapacitors can be classified into two types [6]: pseudo-supercapacitors and electric double-layer supercapacitors (EDLCs). Pseudo-supercapacitors store energy through rapid and reversible intercalation or redox reactions of electroactive species on the electrode surface. By contrast, EDLCs accumulate at the interface between the electrode and the electrolyte through the electrostatic action of the charge. Therefore, EDLCs do not undergo any chemical reaction during charging/discharging, resulting in high power density and cycling stability but low specific capacitance [7]. To enhance the performance and practical applications of supercapacitors, researchers integrated EDLCs with the pseudocapacitor mechanism for the simultaneous utilization of two energy storage mechanisms on the electrode surface, leading to significant improvements in capacitance, energy density, cycle life, and stability [8].

Carbon-based materials, including activated carbon, carbon nanotubes, and graphene, have become popular electrode options given their high specific surface area, superb electrical conductivity, and good cycling stability, making them ideal electrode materials for EDLCs [9,10]. However, heteroatomic doping is also one of the most promising choices in the field. N-doped nanoporous biochars show enhanced electrical conductivity without affecting the basic structure of the raw material [11]. Additionally, the nanoporous structure can be enhanced by increasing the specific surface area, thereby improving the electrical storage performance of the supercapacitors [12]. Further, concerns about environmental and sustainability issues pertaining to non-renewable carbon sources have driven the development of renewable, eco-friendly materials for supercapacitor applications [13,14].

Sourced from renewable and abundant sources such as plants, agricultural waste, and organic materials, biomass-derived carbon materials have shown great potential as sustainable energy storage solutions [15]. As a prominent renewable resource, biomass has become an excellent source of precursors for electrode materials, which entail low preparation costs, abundant resources, and easy handling for environmental protection [16]. Among various biomass-derived carbon materials, algal biomass has attracted significant attention because of its unique characteristics, including rapid growth rates, high photosynthetic efficiency, and minimal competition with food resources [17]. Algae can be cultivated in various environments, including wastewater and saline water, thereby avoiding the use of valuable freshwater resources. In eutrophic water bodies, however, rising concentrations of nutrients such as N and P can lead to the breeding of large numbers of algae and other plankton, causing hazards such as reduced biodiversity, depletion of dissolved oxygen in water bodies, and generation of toxic and harmful substances [18]. As biomass-based porous carbon electrode materials with optimized hetero-electrochemical properties sought after by researchers, algae-based precursors are superb raw materials for porous carbon electrode materials which, in turn, are useful for the secondary utilization of waste materials. Recently, Luo et al. prepared micro/mesoporous carbon from algae (*Kelp*) through a self-template method [19]. Algae containing mineral salts and hydroxyl/carboxyl groups are used as precursors to synthesize the special nanoporous carbon structure, which can significantly improve the performance of electrode materials [20]. *Spirulina*, as a widely available microbial resource, is characterized by rapid growth and wide distribution. The composition of *Spirulina* mainly includes proteins, polysaccharides, and lipids [21]. In particular, *Spirulina* is easily liquefied into water-soluble organics rich in nitrogen functional groups that are similar to soluble organic reagents for nitrogen doping as nitrogen sources [22]. These characteristics make it a promising candidate for new sustainable and low-cost biomass-derived carbon materials for supercapacitors [23].

**Figure 1:**
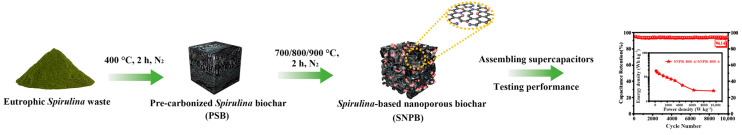
Schematic representation of the formation mechanism for the SNPB.

This work utilized *Spirulina* as a precursor for nanoporous biochar preparation via in situ nitrogen–oxygen heteroatom co-doping through a pre-carbonization coupling pyrolytic activation process for supercapacitor application (Figure 1). The effects of activation temperature and KOH mass ratio on carbon properties were investigated. Given the unique layered porous microstructure and the incorporation of heteroatoms, SNPB-800-4 exhibited high capacitance (348.5 F g^−1^ at a current density of 1 A g^−1^), excellent rate performance (energy of 17.99 W kg^−1^ at a current density of 0.5 A g^−1^), and long-term stability in aqueous electrolytes (94.14% after 10,000 cycles at a current density of 10 A g^−1^ in 6 M KOH). This work demonstrated the feasibility of converting *Spirulina* into N, O-dually doped biochar for high-performance supercapacitors, which can achieve the resourceful use of waste, reduce environmental pollution, and promote sustainable development.

## 2. Materials and Methods

### 2.1. Sample Preparation

*Spirulina* was harvested from Chenghai Lake, Yunnan (China). The raw *Spirulina* was washed multiple times with deionized water and ethanol to remove impurities and dried for 12 h in an oven at 80 °C. *Spirulina*-based nanoporous biochars (SNPBs) were prepared through pre-carbonization and activation using *Spirulina* as a precursor and KOH as an activating agent. Initially, the dried *Spirulina* powder was pre-carbonized in a tubular furnace (400 °C for 2 h, heating rate: 5 °C min^−1^) under N_2_ to remove volatile compounds. Subsequently, the obtained pre-carbonized *Spirulina* biochar (PSB) was mixed with KOH in different mass ratios of 1:1, 1:2, and 1:4. The mixtures were heat-treated at 700 °C, 800 °C, and 900 °C for 2 h in N_2_ flow with a heating rate of 5 °C min^−1^. The obtained product was washed with 1 M HCl and deionized water until the filtrate reached neutrality. The residue was dried at 105 °C for 12 h to obtain the SNPB. The final products were named SNPB-X-Y, where X refers to the activation temperature and Y refers to the KOH ratio.

### 2.2. Structure and Surface Characterization

The microstructures, morphologies, and crystallinity of the samples were characterized by scanning electron microscopy (SEM, Gemini SEM 500, Zeiss, Germany), X-ray diffraction (XRD, D8 ADVANCE, Bruker, Germany, using Cu Kα radiation), and Raman spectrometry (LabRAM HR800, Horiba Jobin Yvon, France, under excitation at 532 nm). Elemental analysis (Vario EL III, Elementar, Germany) and X-ray photoelectron spectroscopy (XPS, ESCALAB 250Xi, Thermo Fischer, Waltham, MA, USA, using Al Kα monochromatic X-ray source) were employed to investigate elemental contents and surface functional groups. The specific surface area (SSA) was calculated using the BET (V-Sorb 2800P, Beijing Guoyi Precision Testing, Beijing, China) method based on adsorption data in the partial pressure (P/P_0_) ranging from 0.02 to 0.25, and the total pore volume was determined from the amount of nitrogen adsorbed at a relative pressure of 0.99. The pore size distributions (PSDs) were calculated from the adsorption isotherms according to nonlocal density functional theory (NLDFT).

### 2.3. Electrochemical Measurements

The electrochemical properties of the SNPBs were initially explored using a three-electrode system in 6 M KOH aqueous solution. To fabricate the electrodes for electrochemical measurements, we mixed the prepared activated biochars with polytetrafluoroethylene (PTFE) and acetylene black at a mass ratio of 8:1:1 in ethanol to form a slurry that was pressed onto a nickel foam current collector (1 × 1 cm^2^). Among them, the SNPB electrode material was composed of 0.008 g of activated biochar, 20 µL of PTFE, and 0.001 g of acetylene black. A platinum foil electrode (as the counter electrode) and a Hg/HgO electrode (as the reference electrode) were employed in a standard three-electrode setup. Cyclic voltammetry (CV), galvanostatic charge–discharge tests (GCD), and electrochemical impedance spectroscopy (EIS) were conducted using a CHI760E Electrochemical Workstation to study the electrochemical property of the samples. EIS was conducted continuously on an electrochemical workstation at an open-circuit voltage with an AC amplitude of 5 mV and a frequency range of 0.1 Hz to 100 kHz. The voltage operating window of CV was set to −1 V to 0 V at different scan rates (5–200 mV s^−1^). The symmetric supercapacitor cells were assembled with circular electrode sheets in a two-electrode system. The cells were tested in the voltage range of 0–1.8 V. Cycle stability tests were performed on a NEWARE battery program control test system.

According to the charge–discharge curves, the specific capacitance of electrodes can be calculated using the following equation [24]:

(1) C=I×Δt /m×ΔV

where *C* (F g^−1^) represents the specific capacitance, *I* (mA) denotes the discharge current, Δ*t* (s) indicates the discharge time, and Δ*V* (V) signifies the discharge voltage range. m (mg) corresponds to the mass loading of active materials in both electrodes.

The energy density *E* (Wh kg^−1^) and power density *P* (W kg^−1^) can be calculated according to the following equations:

(2) $$E=\frac{1}{2\times 3.6}\times C\times {\Delta V}^{2}$$

(3) $$\;P=3600\times \frac{E}{\Delta t}$$

where Δ*V* (V) represents the discharge potential range exclusive of the IR drop, and Δ*t* (s) indicates the discharge time.

## 3. Results and Discussion

### 3.1. Material Characteristics

**Figure 2:**
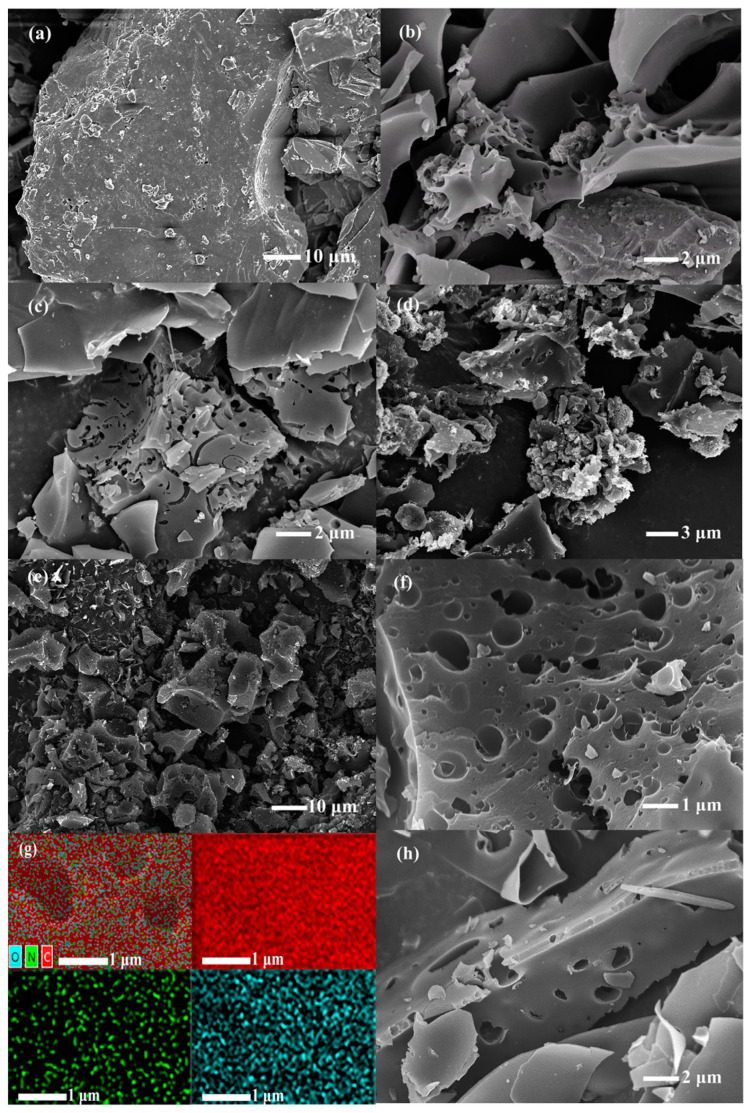
SEM images of (**a**) PSB, (**b**) SNPB-700-4, (**c**) SNPB-800-1, (**d**) SNPB-800-2, and (**e**,**f**) SNPB-800-4. (**g**) EDS mapping of SNPB-800-4 and (**h**) SEM image of SNPB-900-4.

The morphologies of the obtained porous carbons were characterized by SEM. As depicted in Figure 2a, the PSB featured a stacked structure composed of lamellar structures with diameters ranging from tens of micrometers and thicknesses varying from hundreds of nanometers to a few micrometers. Given pyrolysis and the partial removal of organic compounds such as collagen and hemicellulose, the surface of the PSB also presented poor pore structure. Pre-carbonization at a moderate temperature (400 °C) ensured the controlled pyrolytic activation process and a high yield of SNPBs (approximately 13–20%), which was considerably higher than that of one-step activated *Spirulina* (~0.2%) [25,26]. KOH played crucial roles in carbon activation and reacted with carbon as expressed in the following equation [27,28]:

(4) $$6KOH+2C\to 2K+3H_{2}+2K_{2}CO_{3}$$

SEM images (Figure 2b–h) of the SNPBs revealed 3D scaffolding frameworks of porous carbon nanosheets. At an activation temperature of 800 °C, as the activation ratio increased, the looseness of the carbon material was enhanced, and the original irregular microporous structure was transformed into uniform micro/mesopores, especially for SNPB-800-4 (Figure 2f). In addition, many irregular mesh pore structures, which were favorable for fast electron/ion transport [29], were observed. Figure 2g shows the EDS mapping images of SNPB-800-4, which indicated that in situ N, O-dual doping at the surface of SNPB-800-4 was achieved with a uniform distribution. With increased magnification, numerous mesopores became visible, indicating that SNPB-800-4 possessed a multi-level porous structure. The high-density micropores and open cavities of SNPB-800-4 were particularly advantageous for the rapid transportation of electrolyte ions [29,30,31].

**Figure 3:**
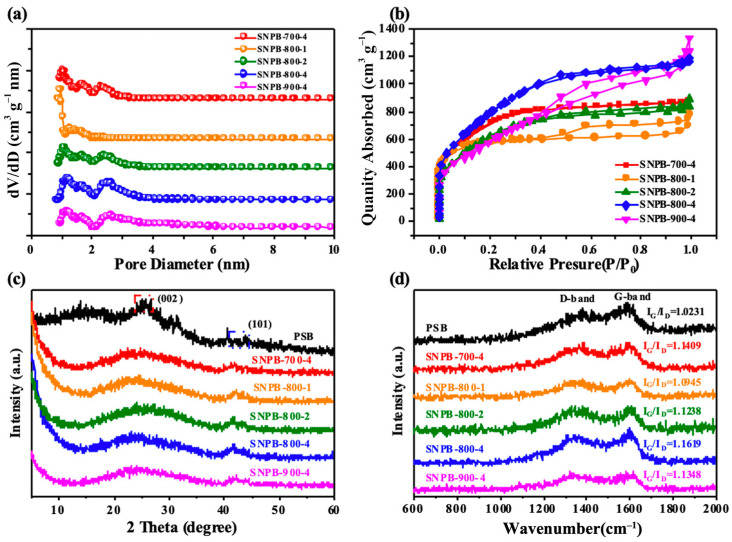
(**a**) Pore size distributions of SNPBs. (**b**) Nitrogen adsorption–desorption isotherms of SNPBs. (**c**) XRD patterns of PSB and SNPBs. (**d**) Raman spectra of PSB and SNPBs.

Table 1 shows the SSA and pore characterizations of all the samples. SNPB-800-4 had well-developed pore structures with ultra-high SSA of 2923.7 m^2^ g^−1^, a huge pore volume of 1.84 cm^3^ g^−1^ (mesopore volume of 1.21 cm^3^ g^−1^), and an average pore width of 3.108 nm. As the activation temperature increased, the number of mesopores that were produced increased, leading to an enlargement in pore volume. Notably, SNPB-900-4 had the smallest SSA, which may be due to the increase in temperature and the increase in KOH erosion leading to the collapse of the carbon skeleton. The porosity of SNPBs became developed with the increase in the mixed mass ratio of KOH and PSB. The micropores were primarily formed at low KOH/PSB mass ratios; however, many mesopores formed at high KOH/PSB mass ratios, leading to a gradual increase in SSA but a noticeable decrease in the proportion of micropores (Figure 3a). Thus, as the KOH/PSB mass ratio increased, more micropores enlarged to mesopores because of the enhanced corrosive pore-creating capacity of KOH.

As shown in Figure 3b, an intermediate behavior between types I and IV reversible isotherms was observed for all SNPBs, indicating the simultaneous presence of micropores and mesopores [32,33]. The dramatic increase in the isotherm at relatively low pressure (P/P_0_ < 0.2) could be attributed to the presence of high-density micropores. At medium relative pressure (P/P_0_ = 0.4–0.9), the presence of a hysteresis loop proved the existence of mesopores. Furthermore, the slight rise at high relative pressure (P/P_0_ = 0.9–1.0) revealed the presence of macropores [34,35]. Further, the H4-type hysteresis loops observed at high pressures implied the formation of micro/mesoporous structures [36,37]. The PSD curves (Figure 3a) calculated using the NLDFT method also revealed that the pore distribution in all SNPBs consisted mainly of micropores (<2 nm), small mesopores (2–4 nm), and large mesopores (4–40 nm). These lamellar porous carbons were preferred because of their high available surface area and well-developed pore network, as well as open ion diffusion channels [38]. Therefore, the SNPBs could be considered hierarchical nanoporous biochars. Charge or electrolyte ion accumulation occurs primarily in micropores, whereas macropores and mesopores are known to facilitate electrolyte transfer to micropores, improving the efficiency of the conduction process [34,35]. Therefore, the hierarchical SNPB has excellent potential as an electrode material for supercapacitor applications.

The crystallinity of biochars is also crucial for their ability to function as a supercapacitor electrode. The XRD spectra of the SNPBs are displayed in Figure 3c. The broad diffraction peaks near 25° and weak peaks near 45° were attributed to the interlayer spacing (002) and planar spacing (100) lattice planes, respectively, which indicated the presence of certain amorphous carbon and graphitized structures in the samples [22]. The relatively weak (101) peaks indicated the uneven graphitization and amorphous carbon characteristics of the samples [39]. Moreover, the patterns of SNPBs presented a significantly enhanced intensity in its small-angle region (2θ < 20°), indicating the presence of high-density micropores, which corresponded to the N_2_ adsorption–desorption isotherm analysis. The weak intensity of the carbons with increasing temperatures also suggested increased defects and disordered structures, as high-temperature activation was conducive to generating numerous large pores and defects [40]. Raman spectroscopy was also employed to investigate the crystallinity of the samples [41]. As illustrated in Figure 3d, G band (1580 cm^−1^; a characteristic feature of graphitic layers) and D band (1350 cm^−1^) were identified in the Raman spectra of all samples [42,43]. The intensity ratios (I_D_/I_G_) of the G and D bands could be used to evaluate the degree of graphitization [40]. The abundance of sp^2^ bonded carbon atoms was observed at I_G_/I_D_ > 1. Given the I_G_/I_D_ values of 1.023 and 1.162 for PSB and SNPB-800-4, respectively, SNPB-800-4 exhibited a slightly high degree of graphitization, which was consistent with the strong in-plane (101) peak presented in the XRD pattern of the SNPBs (Figure 3c) [44]. The I_G_/I_D_ value (1.162) of SNPB-800-4 also suggested that the heteroatomic doping of *Spirulina platensis* itself formed additional pores during graphitization in the carbon skeleton. This feature is believed to be conducive to ion diffusion and beneficial for improving electrochemical performance.

Table S1 (Supporting Information) shows the elemental composition of various samples. The dried *Spirulina* contained 10.22 wt.% N and 36.59 wt.% O, which were significantly higher than those found in lignocellulosic biomass (e.g., wood, straw, and rice husk) [45,46,47]. The N content of PSB was also as high as 10.35 wt.%, which was beneficial for in situ N, O doping in the sequential pyrolytic activation. As the mass ratio of KOH/PSB increased, the N content of the sample also rose. This trend occurred because KOH promoted PSB pyrolysis at high temperatures, leading to other reactions such as dehydrogenation and carbonization, which formed pores in the activated biochar and elevated the N content due to the decreased biochar yields [48]. 

**Figure 4:**
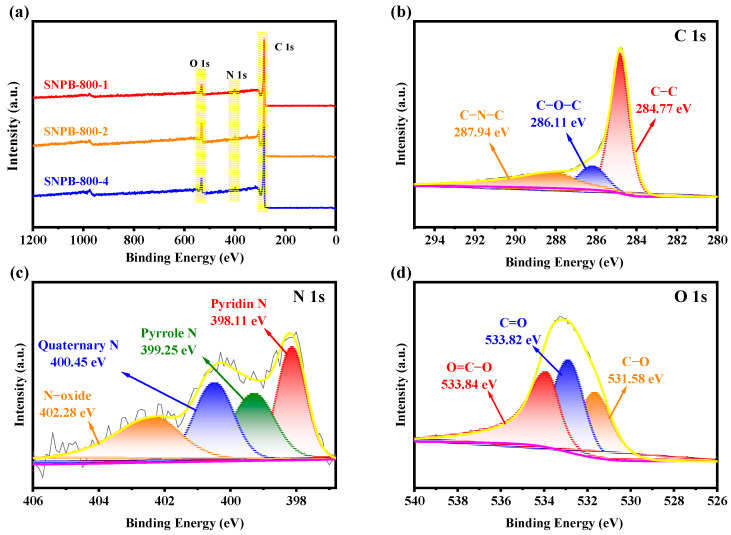
(**a**) XPS full spectrum of SNPB-800-Y. (**b**) XPS high-resolution spectrum of C1s. (**c**) XPS high-resolution spectrum of N1s. (**d**) XPS high-resolution spectrum of O1s.

The chemical composition on the surface of SNPB-800-Y (Y = 1, 2, 4) was characterized by XPS (Figure 4a). The XPS spectrum of SNPB-800-4 revealed the presence of C (~80.58 At.%), O (~13.78 At.%), and N (~2.55 At.%). The high-resolution C 1s spectrum (Figure 4b) of SNPB-800-4 was resolved into three individual peaks, corresponding to C-C (~284.77 eV), C-O-C (~286.11 eV), and C-N-C (~287.94 eV). The high-resolution N 1s spectrum (Figure 4c) was deconvoluted into four peaks located at ~398.11, ~399.25, ~400.45, and ~402.28 eV, corresponding to the pyridine, pyrrole, quaternary, and N-oxide nitrogen groups, respectively. Pyridine and pyrrole are conducive to the formation of the surface and edge defects of biochars, as well as the increase in the active sites for the electrochemical reaction, thereby triggering the pseudocapacitance behavior during the charge–discharge process [49]. Pyridine and pyrrole induced the generation of pseudocapacitance, such as Faraday processes, shown in Equations (5) and (6) [50]:

(5) $$>\;\mathrm{CH}\text{-}NH_{2}+2OH^{-}\;↔\;>\;C=\mathrm{NH}+2H_{2}+2e^{-}\;$$

(6) $$>\;C\text{-}NH_{2}+2OH^{-}\;↔\;>\;C\text{-}\mathrm{NHOH}+H_{2}O+2e^{-}\;$$

Additionally, pyrrole and nitrous oxide groups in the carbon skeleton provided positive charges, altered charge density, reduced charge migration resistance, and improved conductivity [51,52]. The O 1s spectrum (Figure 4d) revealed the presence of three oxygen-based components: C-O (~531.38 eV), C=O (~533.82 eV), and COOR (~533.84 eV). Oxygenated groups enhanced the wettability of biochars to aqueous electrolytes and introduced Faraday pseudocapacitance to improve electrochemical performance [49]. 

The analysis of material characteristics revealed that SNPBs possessed a well-developed hierarchical nanoporous structure with a large SSA, high graphitization, and favorable heteroatom doping. As a result of these properties, SNPBs have good potential as a high-performance electrode material for supercapacitors.

### 3.2. Electrochemical Properties in a Three-Electrode System

**Figure 5:**
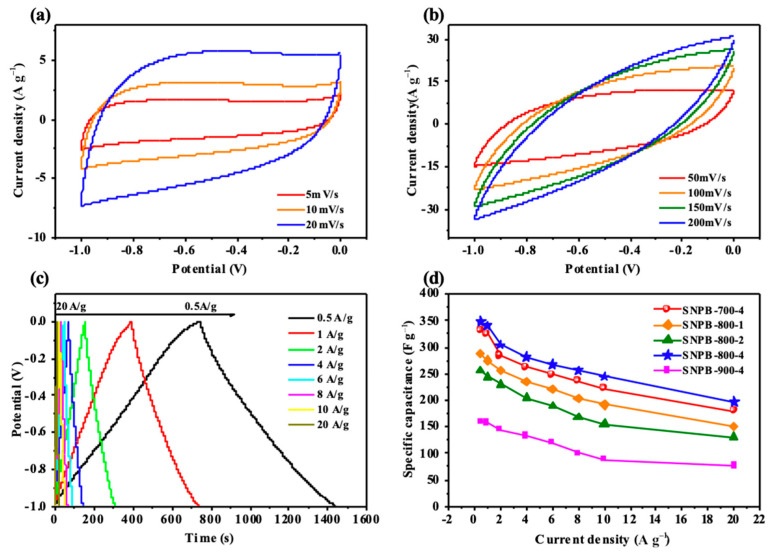
(**a**,**b**) CV curves of SNPB-800-4 at different scanning rates from 5 to 200 mV s^−1^. (**c**) GCD curves of SNPB-800-4 at different densities from 0.5 to 20A g^−1^. (**d**) Specific capacitances of the SNPB electrodes.

The electrochemical performance of SNPBs as electrode materials was first evaluated in a three-electrode system with 6 M KOH as the electrolyte. The cyclic voltammetry (CV) curves were assessed in a potential window from −1 to 0 V with a scan rate of 50 mV s^−1^ (Figure S1, Supporting Information). The CV curves of SNPB-800-4 exhibited the largest area and most rectangular shape, which were characteristic features of typical EDLCs. The CV curves of SNPB-800-4 were investigated at different scan rates (Figure 5a,b). At low scan rates (5–50 mV s^−1^), enhanced diffusion control capacitance was observed because the electrolytic charge could efficiently diffuse to the electrode surface. However, as the scan rate increased from 50 mV s^−1^ to 200 mV s^−1^, the rectangular and symmetric shapes of the CV curves became slightly distorted. This distortion was primarily caused by the limited accessibility of active electrode material particles at high scan rates [6]. The charge–discharge performance of SNPBs prepared under different activation temperatures was investigated at a current density of 1 A g^−1^ (Figure S2, Supporting Information). SNPB-X-4 (X = 700, 800, 900) exhibited almost symmetrical isosceles triangles, indicating good capacitive behavior [41]. SNPB-800-4 had the longest charge–discharge time, which implied its maximum specific capacitance (Equation (1)). Additionally, the charge–discharge performance of SNPB-800-4 was evaluated at a current density range of 0.5–20 A g^−1^ (Figure 5c). The pseudocapacitive behavior of N-containing functional groups in SNPB-800-4 resulted in a constant-current charge–discharge curve that was not a strictly symmetric isosceles triangle. The curve was slightly distorted because of the good reversibility and conductivity of SNPB-800-4. The bending of the charging curve of SNPB-800-4 at high voltage was not remarkable because of the increase in potential affected ion transport [12]. Longer charging and discharging times at low current densities were due to the sufficient time for electrolyte ions to enter and diffuse into the electrode’s pores compared with those at high current densities [15]. This behavior further demonstrated the excellent electrochemical performance of SNPBs in supercapacitor applications.

**Figure 6:**
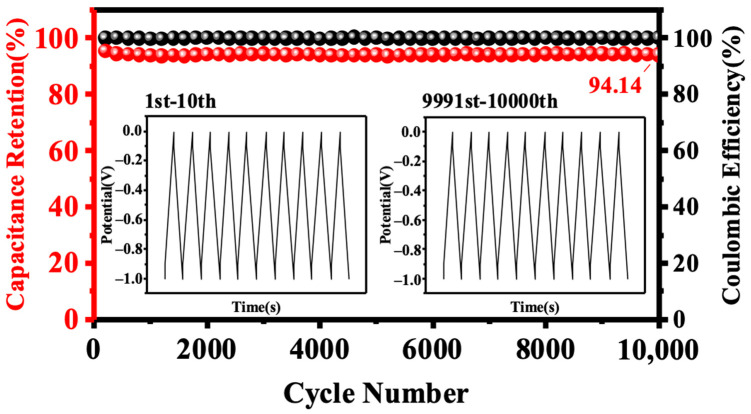
Cycling performance of SNPB-800-4 at 10 A g^−1^ for 10,000 cycles.

As presented in Figure 5d, the specific capacitances of the SNPB electrodes were calculated from the charge–discharge curves at various current densities. Notably, the specific capacitance of SNPB-800-4 reached 348.5 F g^−1^ at a current density of 1 A g^−1^. The ability to maintain excellent specific capacitance even at high current densities serves as evidence of the superior rate performance exhibited by the SNPB-800-4 electrode material. At a current density of 10 A g^−1^, SNPB-800-4 had a capacitance retention of 94.14% after 10,000 cycles (Figure 6), which demonstrated its good cycling stability. The exceptional electrochemical performance of SNPB-800-4 could be attributed to these reasons: (1) the ultra-high SSA (2923.7 m^2^ g^−1^) and total pore volume (1.8448 cm^3^ g^−1^) greatly promoted the diffusion rate of electrolyte ions [53]. The unique hierarchical structure of SNPB-800-4 and optimized PSD provided low resistance paths for fast ion transport, which also greatly promoted electrolyte accessibility to the surface of the micropores. (2) In situ doping of heteroatomic oxygen (13.78%) and nitrogen (2.55%) promoted the formation of active N-doped sites and the generation of pseudocapacitance [54].

EIS spectra in the frequency range of 0.01–10,000 Hz were investigated (Figure S3, Supporting Information) to explore the different resistances associated with the charge storage process and further interpret the behavior of the prepared electrodes. In detail, the Nyquist curve of SNPB-800-4 exhibited a steep linear trend at low frequencies and a small semicircle at high frequencies, indicating almost ideal capacitance characteristics and low equivalent series resistance. This result was attributed to the superior intrinsic electronic properties and more efficient transport of ions through the electrode compared with other electrodes [17].

### 3.3. Electrochemical Properties in a Two-Electrode System

**Figure 7:**
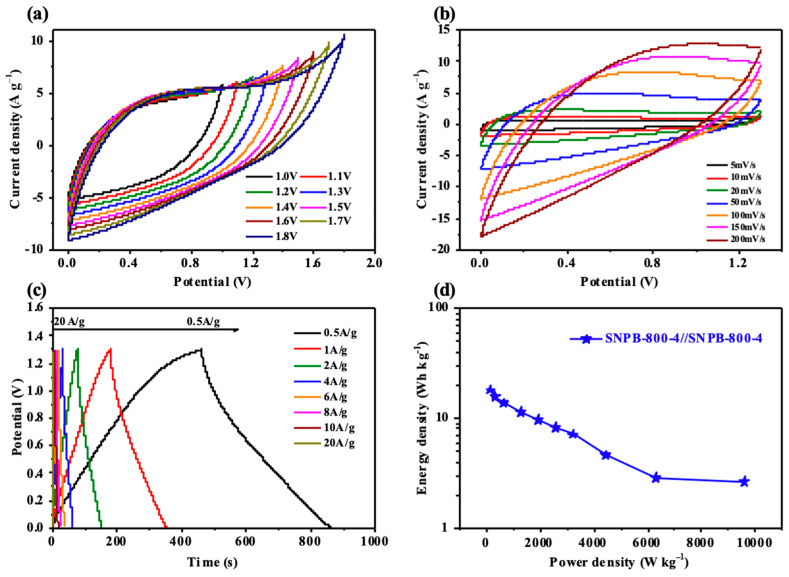
(**a**) CV curves of SNPB-800-4//SNPB-800-4 at different voltage windows of 1 to 1.8 V. (**b**) CV curves of SNPB-800-4/SNPB-800-4 at different scanning rates of 5 to 200 mV s^−1^ under a 1.3 V voltage window. (**c**) GCD curves at different densities from 0.5 to 20 A g^−1^. (**d**) Ragone plot of SNPB-800-4//SNPB-800-4.

SNPB-800-4 was used to assemble a symmetric supercapacitor (SNPB-800-4//SNPB-800-4) using a 6 mol L^−1^ KOH alkaline electrolyte to explore its potential for practical applications. The CV curves of the SNPB-800-4//SNPB-800-4 electrodes at a scan rate of 50 mV s^−1^ maintained a rectangular shape within a voltage range of 0–1.8 V. However, at an expanded voltage window over 1.3 V, the CV curve displayed a “sickle” distortion (Figure 7a). Thus, a voltage window of 0–1.3 V was set to study the electrochemical performance of the symmetric supercapacitor. Quasi-rectangular-shaped CV curves and nearly isosceles triangle GCD curves were observed for the SNPB-800-4 electrode (Figure 7b,c). Notably, a well-maintained rectangular shape was observed even at a high scan rate of 200 mV s^−1^, demonstrating excellent double-layer capacitance behavior (Figure 7b). Additionally, minor IR drops were detected in GCD curves even at a high current density of 20 A g^−1^ (Figure 7c), indicating low internal resistance [51,52]. N, O-dually doped biomass-derived carbon as an electrode material often exhibits significant electron donor characteristics and high charge mobility by providing high surface energy and enhanced surface wettability [55]. The Nyquist plots of the supercapacitor using KOH as an electrolyte were nearly parallel to the imaginary axis in the low-frequency region, providing further evidence for the ideal capacitive performance of the SNPB-800-4 electrode (Figure S4, Supporting Information). Moreover, minor values were observed for the tests on the intrinsic ohmic resistance (R_s_ = 0.76 Ω) and charge transfer resistance (R_ct_ = 1.01 Ω), indicating high electrode conductivity, good electron transfer rates, and rapid diffusion of electrolyte ions in the two-electrode system. An ultra-high specific capacitance of 307 F g^−1^ at a current density of 0.5 A g^−1^, with a retained capacitance of 47 F g^−1^ at a current density of 20 A g^−1^, was observed in the specific capacitance plots of SNPB-800-4//SNPB-800-4 at different current densities (Figure S5, Supporting Information). Promisingly, SNPB-800-4//SNPB-800-4 achieved the maximum energy and power densities of 17.99 W kg^−1^ and 162.48 Wh kg^−1^, respectively, at a current density of 0.5 A g^−1^, and the energy density was maintained at 2.7 Wh kg^−1^ when the power density was increased to 9685.08 W kg^−1^ at a current density of 20 A g^−1^ (Figure 7d). The performances of some recent algal-derived electrode materials reported in the literature are listed in Table 2, among which SNPB-800-4 exhibited great advantages in both SSA and electrochemical properties. For better evaluation of SNPB-800-4 in practical applications, the performance of SNPB-800-4 was also compared with that of commercial carbon materials (Table S2). SNPB-800-4 had excellent SSA and specific capacitance. Therefore, SNPB-800-4 has a strong potential to be used as an electrode material for supercapacitors.

## 4. Conclusions

In summary, in situ N, O-doped hierarchical nanoporous biochars (SNPB-X-Y) were prepared from waste eutrophic *Spirulina* through pre-carbonization coupled with pyrolytic activation using *Spirulina* as the carbon and nitrogen source and KOH as the activator in the N_2_ atmosphere. The effect of pyrolysis temperatures (X = 700 °C, 800 °C, and 900 °C) and activation ratio (Y = 1:1, 1:2, and 1:4) on the physicochemical properties and electrochemical performance of the obtained nanoporous biochars was investigated. SNPB-800-4 exhibited a high SSA of 2923.7 m^2^ g^−1^ and optimal pore size distribution of 1.8448 cm^3^ g^−1^, with mesopores accounting for 65.67%. These favorable characteristics contributed to its excellent energy storage performance, with a specific capacitance of 348.5 F g^−1^ at a current density of 1.0 A g^−1^ in a three-electrode system and 304 F g^−1^ at 0.5 A g^−1^ in a two-electrode system. Moreover, the functionalized biochar demonstrated a high retention rate and impressive cycling stability (with only 5% capacitance loss after 10,000 cycles at 10 A g^−1^). This work demonstrated the ready disposal of waste eutrophic algae *Spirulina* to develop renewable functionalized biochar materials for high-performance energy storage devices. In the subsequent research, the introduction of silicon, phosphorus, or other doping elements into the activated biochar can also alter the chemical composition of the activated biochar and improve its electrochemical properties.

## Figures and Tables

**Table 1:** Pore structure parameters of porous carbon prepared from *Spirulina platensis*.

Sample	S_BET_ ^a^	S_micro_ ^b^	S_meso_ ^c^	V_t_ ^d^	V_micro_^e^	V_meso_ ^f^	V_meso_/V_t_	Pore Size ^g^
	(m^2^ g^−1^)	(m^2^ g^−1^)	(m^2^ g^−1^)	(cm^3^ g^−1^)	(cm^3^ g^−1^)	(cm^3^ g^−1^)	%	(d, nm)
PSB	0.9027	——	2.2235	0.0025	0.0002	0.0028	——	4.772
SNPB-700-4	2515.8	537.4	632.3	1.3606	0.2065	0.4775	35.09	3.021
SNPB-800-1	2096.1	1676.1	327.8	1.2051	0.6969	0.4385	36.39	5.351
SNPB-800-2	2255.6	209.8	875.3	1.3788	0.0302	0.7421	53.82	3.391
SNPB-800-4	2923.7	——	1559.1	1.8448	——	1.2114	65.67	3.108
SNPB-900-4	2178.3	——	1710.9	2.0687	——	1.8981	91.75	4.297

^a^ BET surface area; ^b^ micropore (micro < 2 nm) surface area calculated; ^c^ mesopore (2 nm < meso < 50 nm) surface area calculated; ^d^ total pore volume obtained at P/P_0_ = 0.99; ^e^ micropore volume calculated by t-plot method; ^f^ mesopore volume calculated by BJH method; ^g^ average aperture value.

**Table 2:** Comparison of SSA and electrochemical properties between SNPB-800-4 and other algae-derived electrode materials.

Electrode Material	Specific Surface Area (m^2^ g^−1^)	Specific Capacitance (F g^−1^)	Current Density (A g^−1^)	Energy Density (Wh kg^−1^)	Number of Cycles	Cycle Stability (%)	References
SNPB-800-4	2923.7	348.5	1	15.6	10,000	94.14	——
*Enteromorpha prolifera*	2000	200	1	7	10,000	96	[56]
*Kelp*	4425	277	0.1	8	20,000	92	[19]
*Chlorella vulgaris*	3516	142	1	8.9	2200	91.5	[57]
*Ascophyllum nodosum*	1493	207.3	0.5	—	2500	92.3	[58]
*Turbinaria conoides*	173.8	416	1	52	5000	85.3	[59]

## Data Availability

Data sharing not applicable.

## Supplementary Materials

The following supporting information can be downloaded at: https://www.mdpi.com/article/10.3390/nano13172431/s1, Figure S1: CV curves of SNPBs at a 50 mV s^−1^ scanning rate. Figure S2: GCD curves of SNPB-X-4 at a current density of 1 A g^−1^. Figure S3: Nyquist plots of SNPBs. Figure S4: Nyquist plots of SNPB-800-4//SNPB-800-4. Figure S5: Specific capacitance of the SNPB-800-4//SNPB-800-4 electrode at different current densities. Table S1: Element analysis of porous carbon prepared from *Spirulina* platensis. Table S2: Comparison of the performance between SNPB-800-4 and commercial carbon materials. References [60,61,62,63] are cited in the supplementary materials.
