# Supplementary material for: In Situ N, O-Dually Doped Nanoporous Biochar Derived from Waste Eutrophic Spirulina for High-Performance Supercapacitors
Source: Nanomaterials (Basel). 2023 Aug 27;13(17):2431. doi: 10.3390/nano13172431 (PMC10489722; doi:10.3390/nano13172431)
Supplement: Supplementary file 1 [file nanomaterials-13-02431-s001.zip › nanomaterials-2538993-supplementary.pdf]

Supporting Information

In Situ N, O-Dually Doped Nanoporous Biochar Derived from Waste Eutrophic *Spirulina* for High-Performance Supercapacitors

Yihao Geng [1], Jieni Wang [1,2], Xuanyu Chen [1], Qizhao Wang [1], Shuqin Zhang [1,2], Yijun Tian [1,2], Chenxiao Liu [1,2], Lin Wang [1], Zhangdong Wei [1], Leichang Cao [1,2,*], Jinglai Zhang [2] and Shicheng Zhang [3]

[1] Miami College, Henan University, Kaifeng 475004, China; gyh618@henu.edu.cn (Y.G.); jieniwang@126.com (J.W.); xuanyuchen@henu.edu.cn (X.C.); 17367066458@163.com (Q.W.); zhangshuqin@henu.edu.cn (S.Z.); 104753200976@henu.deu.cn (Y.T.); lcxdxyyx@henu.edu.cn (C.L.); wanglin@henu.edu.cn (L.W.); 15565139820@163.com (Z.W.)

[2] College of Chemistry and Molecular Sciences, Henan University, Kaifeng 475004, China; zhangjinglai@henu.edu.cn

[3] Shanghai Key Laboratory of Atmospheric Particle Pollution and Prevention (LAP3), Department of Environmental Science and Engineering, Fudan University, Shanghai 200433, China; zhangsc@fudan.edu.cn

* Correspondence: clch666@henu.edu.cn

Number of Pages: 7
Number of Figures: 5
Number of Table: 2

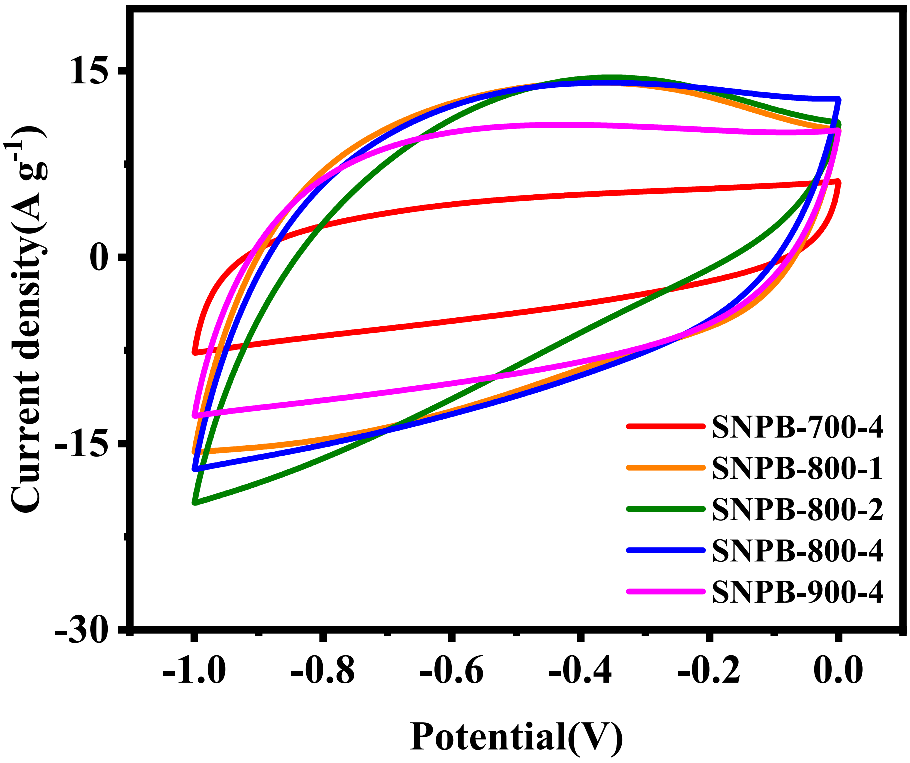

Figure S1. CV curves of SNPBs at 50 mV s^{-1} scanning rate.

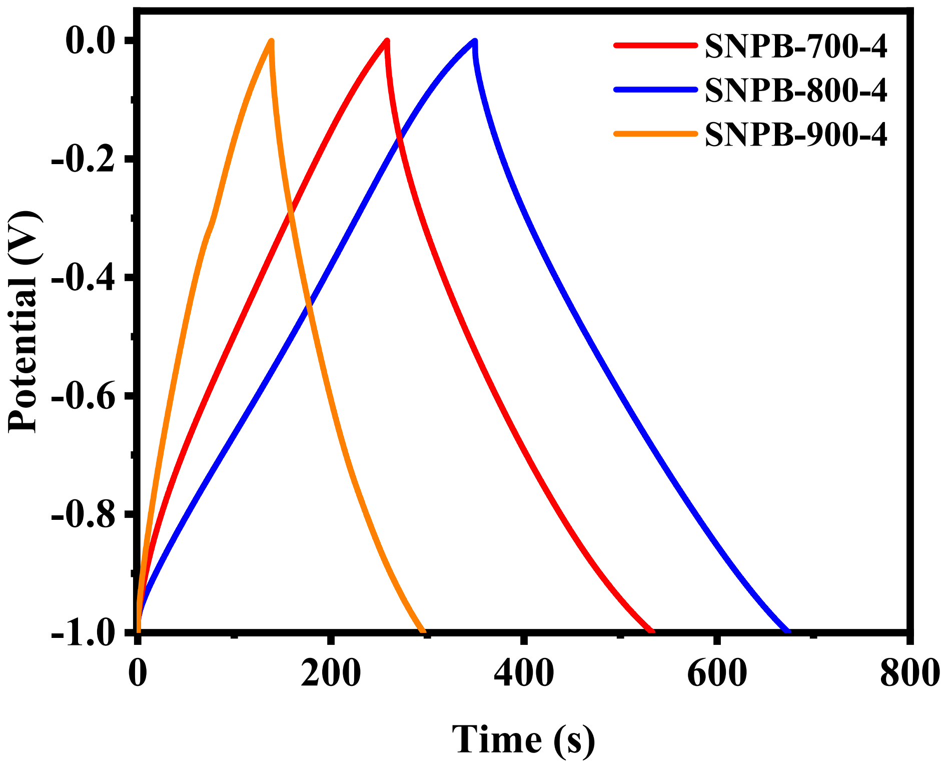

Figure S2. GCD curves of SNPB-X-4 at a current density of 1A g^{-1}.

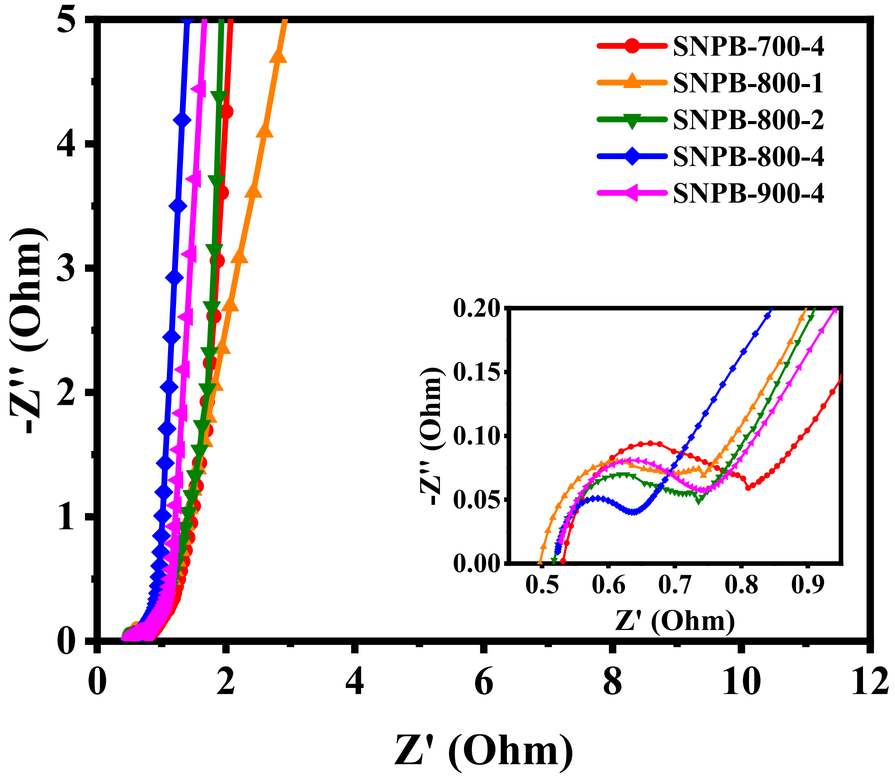

Figure S3. Nyquist plots of SNPBs.

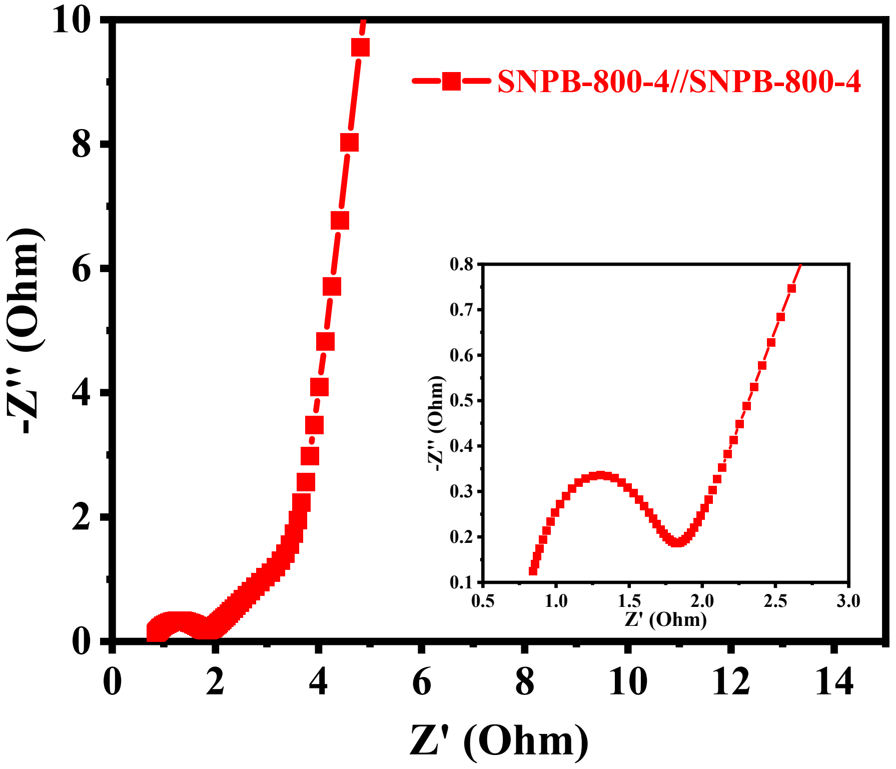

Figure S4. Nyquist plots of SNPB-800-4//SNPB-800-4.

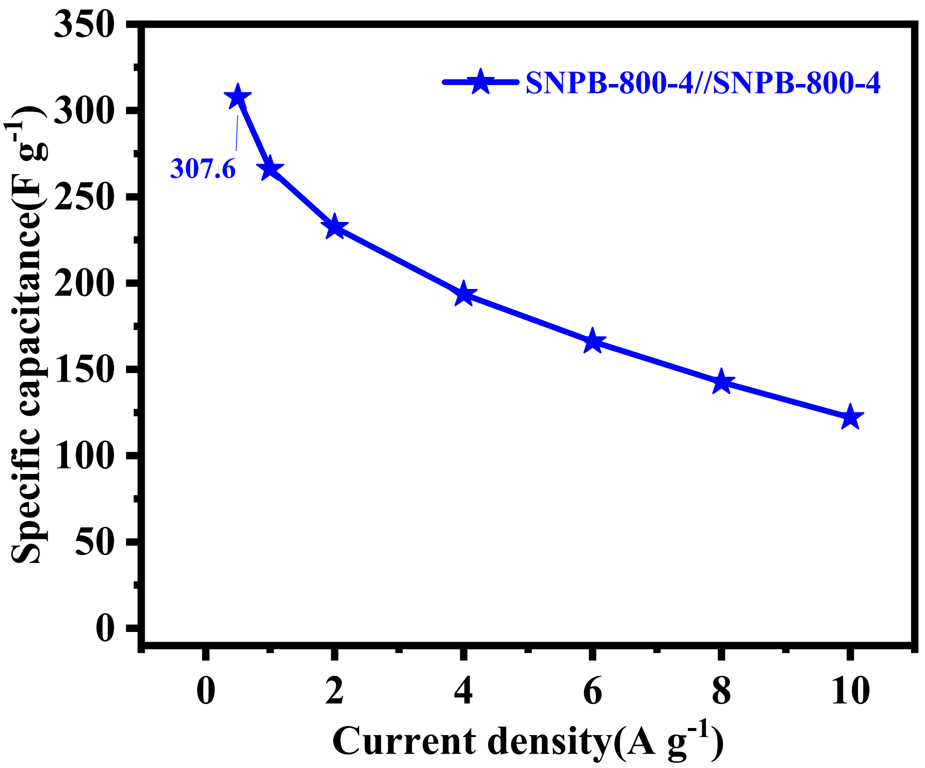

Figure S5. Specific capacitance of the SNPB-800-4//SNPB-800-4 electrode at different current densities.

Table S1. Element analysis of porous carbon prepared from *Spirulina* platensis.

Sample	C (%)	H (%)	N (%)	O (%)	C/N ratio	C/H ratio	C/O ratio	C/(O+N) ratio
Spirulina	45.43	7.76	10.22	36.59	4.45	5.85	1.24	0.97
PSB	59.28	3.84	10.35	26.53	5.73	15.44	2.23	1.61
SNPB-700-4	72.08	3.21	3.42	21.29	21.08	22.45	3.39	2.92
SNPB-800-1	68.73	1.09	1.27	28.91	54.12	63.06	2.38	2.28
SNPB-800-2	75.06	2.91	1.92	20.11	39.09	25.79	3.73	3.41
SNPB-800-4	80.58	3.09	2.55	13.78	31.6	26.08	5.85	4.93
SNPB-900-4	81.11	1.21	2.11	15.57	38.44	67.03	5.21	4.59

Table S2. Comparison of the performance between SNPB-800-4 and commercial carbon materials.

Electrode material	Specific surface area (m^2 g^{-1})	Specific capacitance (F g^{-1})	Current density (A g^{-1})	Number of cycles	Cycle stability (%)	References
SNPB-800-4	2923.7	348.5	1	10,000	94.14	——
Carbon nanotubes	——	231.2	1	10,000	87.3	[1]
Carbon dots	1246	350	1	10,000	92	[2]
Carbon fiber@graphene	2035	552.8	0.1	200	90	[3]
Porous graphene	2160	210	1	5000	94.7	[4]

References:

1. Zhang, Z.; Bi, L.; Tian, Q.; Gao, J.-S.; He, Y., Construction of nickel/cobalt encapsulated by N-doped carbon nanotubes for high performance supercapacitors. Diamond and Related Materials 2023, 110237.
2. Pu, J.; Wang, L.; Huang, J.; Chen, Q.; Jin, Y.; Chen, J., High-performance supercapacitors based on porous activated carbon derived from carbon dots by directly pyrolyzing industrial glucose. Diamond and Related Materials 2023, 132, 109684.
3. Xie, Q.; Bao, R.; Xie, C.; Zheng, A.; Wu, S.; Zhang, Y.; Zhang, R.; Zhao, P., Core-shell N-doped active carbon fiber@graphene composites for aqueous symmetric supercapacitors with high-energy and high-power density. Journal of Power Sources 2016, 317, 133-142.
4. Zheng, C.; Zhou, X.; Cao, H.; Wang, G.; Liu, Z., Synthesis of porous graphene/activated carbon composite with high packing density and large specific surface area for supercapacitor electrode material. Journal of Power Sources 2014, 258, 290-296.
